# PLA-THF-PEG nanoparticles Co-encapsulating AV3 and KH3 for synergistic pancreatic cancer therapy via stromal remodeling and metabolic inhibition

**DOI:** 10.3389/fphar.2026.1723694

**Published:** 2026-02-04

**Authors:** Xiaorong Liu, Yuanmin Hu, Jiaqi Yu, Yuzhe Xue, Xuesong He, Fenfen Jiang

**Affiliations:** 1 Department of Hepatobiliary Surgery, The Second Affiliated Hospital of Jiaxing University, Jiaxing, China; 2 Intensive Care Unit (ICU), The Second Affiliated Hospital of Jiaxing University, Jiaxing, China; 3 Jiaxing University Master Degree Cultivation Base, Zhejiang Chinese Medical University, Hangzhou, Zhejiang, China; 4 Department of Geriatrics, The Second Affiliated Hospital of Jiaxing University, Jiaxing, China

**Keywords:** co-encapsulating, ITGA5 antagonist, pancreatic cancer, PGAM1 inhibitor, pH-sensitive nanoparticles

## Abstract

**Introduction:**

Pancreatic ductal adenocarcinoma (PDAC) is one of the most lethal malignancies, characterized by dense desmoplastic stroma, metabolic reprogramming, and poor therapeutic response. Stromal desmoplasia limits drug penetration, while glycolytic adaptation supports tumor progression. Effective strategies that simultaneously target stromal barriers and tumor metabolism are urgently needed.

**Methods:**

To overcome these challenges, we developed a dual-targeted, pH-responsive nanoplatform (PLA–THF–PEG/AV3/KH3 nanoparticles, termed AKNPs) that co-delivers AV3, a peptide antagonist of integrin α5 (ITGA5), and KH3, an allosteric inhibitor of phosphoglycerate mutase 1 (PGAM1). AKNPs were prepared by self-assembly and characterized for morphology, colloidal stability, and pH-triggered disassembly under acidic conditions mimicking tumor and endo/lysosomal environments. In vitro and in vivo therapeutic evaluations were performed using PANC-1 models.

**Results:**

AKNPs exhibited uniform spherical morphology, excellent colloidal stability, and pH-sensitive disassembly. In vitro, AKNPs showed superior cytotoxicity in PANC-1 cells compared with free AV3 or AV3+KH3, accompanied by downregulation of fibrosis markers (α-SMA, COL1A1), metabolic regulator PGAM1, ITGA5, and immune checkpoint PD-L1, indicating combined stromal-remodeling and immunomodulatory effects. In vivo, in a PANC-1 xenograft model, AKNPs significantly inhibited tumor growth compared with PBS, AV3, or AV3+KH3. Tumor volumes and weights were reduced, and histological analyses revealed decreased fibrosis, lower tumor density, and enhanced necrosis. Western blot analysis of tumor tissues confirmed suppression of α-SMA, COL1A1, ITGA5, PGAM1, and PD-L1.

**Discussion:**

These findings demonstrate that pH-sensitive AKNPs integrate stromal remodeling and metabolic inhibition to synergistically suppress PDAC progression. This nanoplatform provides a promising therapeutic strategy for overcoming stromal and metabolic barriers in pancreatic cancer and may serve as a foundation for future combination regimens.

## 1 Introduction

Pancreatic ductal adenocarcinoma (PDAC) accounts for over 90% of pancreatic cancer cases and represents the fourth leading cause of cancer-related mortality globally. Despite advances in surgical techniques and systemic therapies, the prognosis remains dismal, with a 5-year survival rate of only 7%–9% (Siegel et al., 2023; McGuigan et al., 2018). Pancreatic cancer is often referred to as a “metabolic disease” because of its profound dependence on altered metabolic pathways to sustain proliferation and survival (Ying et al., 2012). Due to the pancreas´ deep retroperitoneal location and the insidious onset of disease, most pancreatic cancers are diagnosed at advanced stages, lacking specific early clinical features (Mizrahi et al., 2020). Current treatment strategies, including gemcitabine- or FOLFIRINOX-based chemotherapy, are limited by systemic toxicity, rapid clearance, and inadequate intra-tumoral drug delivery caused by the dense fibrotic stroma (Conroy et al., 2011).

Current guidelines, such as those from the National Comprehensive Cancer Network (NCCN) and the American Society of Clinical Oncology (ASCO), recommend neoadjuvant therapy followed by surgical resection for patients with borderline resectable or locally advanced PDAC (Tempero et al., 2021; Sohal et al., 2020). However, the clinical value of neoadjuvant chemotherapy remains debated due to the lack of sufficiently powered randomized controlled trials (RCTs), and outcomes vary depending on patient selection and treatment regimens (Versteijne et al., 2022). In addition, there is an inherent risk of tumor progression or early metastasis during systemic treatment in patients who are insensitive to chemotherapy (O'Reilly et al., 2020). These limitations highlight the urgent need for novel therapeutic approaches capable of overcoming the stromal and metabolic barriers inherent to PDAC.

Metabolic reprogramming has emerged as a hallmark of PDAC (Pavlova and Thompson, 2016). Among the metabolic enzymes implicated, phosphoglycerate mutase 1 (PGAM1) plays a critical role in glycolysis by regulating glucose utilization, ATP generation, macromolecular biosynthesis, and redox balance, thereby promoting tumor growth and survival (Hitosugi et al., 2012; Israelsen and Vander Heiden, 2015). Given its central position in metabolic control, PGAM1 represents an attractive therapeutic target (Yang et al., 2022). Recent studies have revealed that inhibition of PGAM1 not only impairs tumor metabolism but also remodels the immune microenvironment and enhances PD-1 blockade efficacy (Zhang et al., 2024). Wen et al. identified KH3, a potent allosteric PGAM1 inhibitor, which significantly suppresses PDAC cell proliferation by reducing glycolysis and mitochondrial respiration, while exhibiting high selectivity and low toxicity (Wen et al., 2019). Nevertheless, the therapeutic potential of KH3 is largely hindered by its limited tumor penetration and inefficient accumulation within the dense desmoplastic stroma characteristic of PDAC.

The pancreatic tumor microenvironment is dominated by cancer-associated fibroblasts (CAFs), primarily derived from pancreatic stellate cells (PSCs). Activated PSCs secrete growth factors and cytokines that promote tumor angiogenesis, invasion, and metastasis (Öhlund et al., 2014). In normal pancreatic tissue, PSCs remain quiescent with abundant vitamin A-containing lipid droplets; however, once activated, they acquire a myofibroblast-like phenotype expressing α-smooth muscle actin (α-SMA) and type I collagen (COL1A1) (Apte et al., 1998; Erkan et al., 2012; Bachem et al., 2005). Integrin α5 (ITGA5) is highly upregulated in activated PSCs, and its blockade has been shown to suppress desmoplasia, decompress tumor vasculature, and enhance chemotherapeutic delivery (Kuninty et al., 2019). The peptidomimetic AV3, a selective ITGA5 antagonist, was previously demonstrated to inhibit PSC activation, attenuate fibrosis, and potentiate gemcitabine efficacy in PDAC models (Kuninty et al., 2018).

However, monotherapies targeting either metabolism or stroma often yield limited efficacy due to the complex interplay between metabolic adaptation and fibrotic remodeling. Therefore, a rationally designed co-delivery system that simultaneously targets both stromal fibrosis and metabolic reprogramming could produce a synergistic therapeutic effect.

Nanotechnology-based drug delivery systems offer a promising avenue to enhance therapeutic outcomes by improving tumor-specific accumulation and minimizing systemic toxicity (Zhang et al., 2019; Yang et al., 2025). In particular, pH-responsive nanoparticles exploit the mildly acidic tumor microenvironment (pH 6.5–6.8) for selective drug release, while remaining stable under physiological conditions (pH 7.4). PLA–THF–PEG amphiphilic copolymers, containing acid-labile tetrahydrofuran (THF) linkages, can self-assemble into micelles with a hydrophobic PLA core and hydrophilic PEG shell, providing an ideal nanocarrier platform for dual-drug encapsulation and controlled, tumor-triggered release (Feng et al., 2018; Chu et al., 2022; Zhu et al., 2014).

**SCHEME 1:**
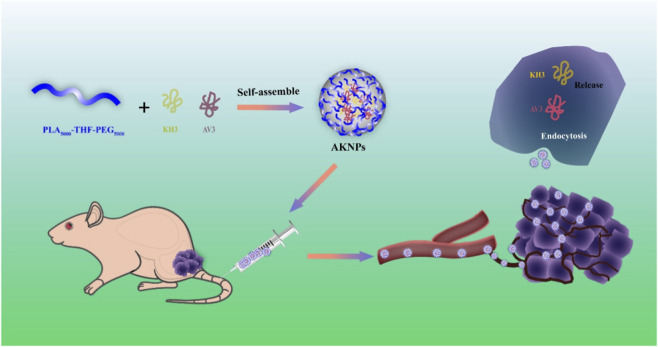
Schematic illustration of the preparation and therapeutic mechanism of AKNPs. PLA_5000_-THF-PEG_5000_ copolymers co-encapsulate AV3, an ITGA5-targeting antifibrotic peptide, and KH3, a PGAM1 inhibitor disrupting glycolytic metabolism, via a self-assembly process to form pH-sensitive nanoparticles (AKNPs). After intravenous administration, AKNPs circulate in the bloodstream and preferentially accumulate in pancreatic tumor tissues. Following endocytosis by tumor cells and exposure to the acidic tumor microenvironment, AKNPs disassemble and release AV3 and KH3, thereby inducing stromal remodeling, metabolic inhibition, and enhanced antitumor efficacy.

In this study, we designed a dual-targeting, pH-sensitive nanoplatform composed of PLA–THF–PEG nanoparticles co-encapsulating AV3 and KH3 (termed AKNPs). As illustrated in Scheme 1, PLA–THF–PEG copolymers were combined with KH3 and AV3 through a self-assembly process to form AKNPs with uniform nanostructure and high encapsulation efficiency. Upon intravenous injection, AKNPs circulate stably in the bloodstream and preferentially accumulate in pancreatic tumor tissues. After endocytosis by tumor cells and exposure to the acidic tumor microenvironment, AKNPs undergo disassembly, triggering the sequential release of AV3 and KH3. The released AV3 suppresses stromal fibrosis by blocking ITGA5-mediated PSC activation, while KH3 inhibits glycolytic metabolism via PGAM1 inhibition, jointly leading to stromal remodeling, metabolic suppression, and enhanced antitumor efficacy.

Collectively, this work aimed to (i) characterize the physicochemical properties and pH sensitivity of AKNPs, (ii) investigate their cellular uptake, cytotoxicity, and mechanistic effects *in vitro*, and (iii) evaluate their therapeutic efficacy and biosafety in PDAC xenograft models. Our results demonstrate that this dual-targeting, pH-sensitive co-delivery system represents a promising strategy to overcome the stromal and metabolic resistance of pancreatic cancer, offering new insights for combinatorial nanomedicine against desmoplastic tumors.

## 2 Materials and methods

### 2.1 Cell lines and cell culture

The human pancreatic cancer cell line PANC-1 was obtained from Zhong Qiao Xin Zhou Biotechnology Co., Ltd. (Shanghai, China). Cells were maintained in high-glucose Dulbecco’s modified Eagle’s medium (DMEM; Zhong Qiao Xin Zhou, China) supplemented with 10% fetal bovine serum (FBS; Zhong Qiao Xin Zhou, China) and 1% penicillin–streptomycin (PS; Zhong Qiao Xin Zhou, China). Cultures were incubated at 37 °C in a humidified atmosphere containing 5% CO_2_.

### 2.2 Synthesis of PLA-THF-PEG copolymer

The pH-sensitive amphiphilic copolymer PLA-THF-PEG was synthesized through a multistep procedure involving esterification, nucleophilic substitution, reduction, and coupling reactions, as previously reported with modifications (Zhu et al., 2014). Briefly, 4-pentenoic acid was oxidized and esterified to obtain the activated intermediate, which was further converted to a nitrophenyl ester. Subsequent reduction with DIBAL-H yielded the hydroxylated intermediate, which was conjugated with PEG_5000_ under acid catalysis. The nitro group was removed by catalytic hydrogenation, and the amine-terminated PEG derivative was finally coupled with PLA_5000_ (succinic anhydride functionalized) using HATU/DIPEA in DMF. The final product (PLA_5000_-THF-PEG_5000_) was purified by column chromatography and obtained as a pale-yellow powder. Its structure was confirmed by ^1^H NMR and FT-IR spectroscopy, which showed characteristic peaks corresponding to PLA, THF, and PEG segments.

### 2.3 Synthesis of AV3 peptide (Ac-RYYRITY)

The AV3 peptide (sequence: Ac-RYYRITY) was synthesized using standard Fmoc-based solid-phase peptide synthesis (SPPS) on Wang resin. Briefly, the procedure involved resin swelling, removal of the Fmoc protecting group, coupling of the first amino acid to the resin, capping of unreacted sites, iterative chain elongation through sequential amino acid coupling, N-terminal acetylation, and final cleavage from the resin. The crude peptide was purified by preparative liquid chromatography and lyophilized to obtain the final product.

### 2.4 Synthesis of KH3

KH3 was synthesized through a four-step procedure starting from 1,2-dihydroxyanthraquinone.

Synthesis of intermediate 2: 1,2-dihydroxyanthraquinone (5 g, 20.8 mM) was dissolved in 350 mL of acetic acid, and nitric acid (1.5 mL, 33.3 mM) was added dropwise at 50 °C. After cooling to room temperature, the crude product was collected by filtration to afford intermediate 2 as a yellow solid (4.15 g, 70%).

Synthesis of intermediate 3: A suspension of intermediate 2 (1.75 g, 6.14 mM) in ethanol (350 mL) was treated with Sn (10.5 g, 341 mM), SnCl_2_·2H_2_O (12.5 g, 55.4 mM), and concentrated HCl (50.4 mL, 604.8 mM). The reaction mixture was stirred overnight at room temperature, concentrated under reduced pressure, and poured into 1 L of water to yield a red precipitate. The crude intermediate 3 was collected by filtration and vacuum dried to give a black solid (1.41 g, 90%).

Synthesis of intermediate 4: Intermediate 3 (255 mg, 1 mmol) was dissolved in anhydrous pyridine (5 mL), and 4-(dimethylamino)benzenesulfonyl chloride was added. The mixture was stirred at room temperature for 4 h and then poured into 10% aqueous HCl (50 mL). The product was extracted with ethyl acetate, washed with brine, dried over Na_2_SO_4_, and purified by silica gel chromatography to afford intermediate 4 as a red solid (1.17 g, 60%).

Final synthesis of KH3: Intermediate 4 (52.1 mg, 0.1 mmol) was combined with tris(dibenzylideneacetone)dipalladium (9.2 mg, 0.01 mmol), BINAP (9.3 mg, 0.015 mmol), and sodium tert-butoxide (48.1 mg, 0.5 mmol) in piperidine (1 mL). The reaction was stirred at 85 °C overnight, cooled, and quenched with 10% aqueous HCl (20 mL). The mixture was extracted with ethyl acetate, washed with brine, dried, and purified by silica gel chromatography to afford KH3 as a yellow solid (27.8 mg, 60%).

The structure of KH3 was confirmed by ^1H^ NMR, ^13C^ NMR, and ESI-MS.

### 2.5 Preparation of nanoparticles

AV3 and KH3 were dissolved with PLA-THF-PEG in DMSO (10 mg/mL). The solution was added dropwise (1 mL/10 min) into 8 mL ultrapure water under stirring. After 1 h, the mixture was transferred into a dialysis bag (MWCO 2,000 Da) and dialyzed for 48 h against water (changed every 4 h). The final nanoparticles were lyophilized into powder.

### 2.6 Nanoparticle characterization

#### 2.6.1 Molecular weight determination

The molecular weight of the PLA-THF-PEG copolymer was determined by gel permeation chromatography (GPC) using an Agilent 1,260 system equipped with a 7.5 × 300 mm, 10 μm GPC column. Tetrahydrofuran (THF) was used as the eluent at a flow rate of 1.0 mL/min, with the column temperature maintained at 35 °C. Polystyrene standards were used for calibration.

#### 2.6.2 Morphological analysis

The morphology of PLA-THF-PEG/AV3/KH3 nanoparticles was examined by transmission electron microscopy (TEM). Briefly, nanoparticles were dispersed in anhydrous ethanol at a concentration of 3 mg/L and sonicated to achieve uniform suspension. A drop of the suspension was placed on a copper grid and air-dried before imaging.

#### 2.6.3 Particle size and zeta potential

The hydrodynamic diameter and zeta potential of PLA-THF-PEG/AV3/KH3 nanoparticles were measured by dynamic light scattering (DLS) using a laser particle size analyzer. Samples were prepared by dispersing the nanoparticles in deionized water, followed by sonication prior to measurement.

#### 2.6.4 pH-triggered release

The pH-responsive release of AV3 and KH3 from PLA-THF-PEG nanoparticles was investigated using a dialysis method. Briefly, drug-loaded nanoparticles were dispersed in phosphate-buffered saline (PBS) and transferred into dialysis bags (molecular weight cutoff: 2000 Da). The bags were immersed in 200 mL of release medium at either pH 7.4 (physiological condition) or pH 5.0 (acidic tumor microenvironment), and incubated at 37 °C with gentle stirring. At predetermined time intervals, 2 mL of the external release medium was withdrawn and replaced with an equal volume of fresh medium to maintain sink conditions. The concentration of KH3 in the release medium was quantified using a fluorescence spectrophotometer (Ex/Em = 401/660 nm), while AV3 content was determined by UV–Vis spectrophotometry. The cumulative release percentage was calculated and plotted as a function of time.

#### 2.6.5 Encapsulation efficiency (EE%) and drug loading (DL%) of AV3 and KH3

The encapsulation efficiency (EE%) and drug loading (DL%) of AV3 and KH3 in AKNPs were determined after nanoparticle preparation and purification. Briefly, free (non-encapsulated) drugs were removed during the dialysis step (MWCO 2,000 Da) as described above, and the purified nanoparticles were collected and lyophilized. To quantify the loaded drugs, an accurately weighed amount of freeze-dried AKNPs (e.g., 2–5 mg) was completely dissolved in DMSO (or DMSO/water mixture) and sonicated if necessary to ensure full disruption of the nanostructure and complete drug extraction. After centrifugation (to remove any insoluble residue), the supernatant was used for drug quantification.

AV3 content was measured by UV-Vis spectrophotometry using an external calibration curve of AV3 prepared in the same solvent system. KH3 content was quantified using a fluorescence spectrophotometer (Ex/Em = 401/660 nm) based on a KH3 standard curve prepared under identical conditions. All measurements were performed in triplicate (n = 3). Encapsulation efficiency and drug loading were calculated as follows:

$$EE\left(\%\right)=\frac{\text{Amount}\;\text{of}\;\text{drug}\;\text{encapsulated}\;\text{in}\;\text{nanoparticles}}{T\text{otal}\;a\text{mount}\;\text{of}\;\text{drug}\;\text{initially}\;\text{added}}$$

$$DL\left(\%\right)=\frac{\text{Mass}\;\text{of}\;\text{drug}\;\text{encapsulated}\;\text{in}\;\text{nanoparticles}}{T\text{otal}\;\text{weight}\;\text{of}\;\text{drug}-\text{loaded}\;\text{nanoparticles}}$$

The resulting EE% and DL% values for both AV3 and KH3 are summarized in Table 1.

**TABLE 1:** Encapsulation efficiency (EE%) and drug loading (DL%) of AV3 and KH3 in AKNPs.

Drug	Encapsulation efficiency (%)	Drug loading content (%)	n
KH3	58.11 ± 1.52	12.6 ± 0.4	3
AV3	85.87 ± 2.55	21.8 ± 0.7	3

### 2.7 Cell viability assay (CCK-8)

Cell viability was evaluated using the Cell Counting Kit-8 (CCK-8, Beyotime, China) according to the manufacturer’s protocol. PANC-1 cells were seeded in 96-well plates at a density of 5 × 10^3^ cells per well in 100 μL of complete medium and cultured overnight to allow adherence. Cells were treated with various concentrations of AV3 (0–80 μM) or KH3 (0–40 μM) for 48 h. To evaluate the effect of the nanoparticle system, cells were divided into four groups: PBS (control), AV3, AV3 + KH3, and AKNPs. After treatment, 10 μL of CCK-8 solution was added to each well and incubated for 1–4 h at 37 °C. The absorbance at 450 nm was recorded using a microplate reader (Thermo Fisher Scientific, United States).

### 2.8 Western blot analysis

Protein expression was quantified by Western blot. PANC-1 cells were lysed on ice in RIPA buffer supplemented with 1% Triton X-100 and 0.2 mM PMSF for 30 min, followed by centrifugation at 12,000 rpm for 15 min at 4 °C to collect the supernatant. Protein concentration was measured with a BCA kit (Beyotime, China). Equal amounts of protein were mixed with 5× SDS loading buffer, boiled at 100 °C for 5 min, and separated by SDS–PAGE. Proteins were transferred to PVDF membranes (Millipore, United States) at 100 V under cold conditions. Membranes were blocked with 5% (w/v) non-fat milk in TBST for 1 h at room temperature and incubated overnight at 4 °C with primary human antibodies (Proteintech, China) as follows: anti-α-smooth muscle actin, (α-SMA, 1:1,000); type I collagen (COL1A1, 1:1,000); PD-L1 (1:1,000); ITGA5 (1:1,000); PGAM1 (1:1,000); GAPDH (loading control, 1:5,000). After TBST washes three times, membranes were incubated with HRP-conjugated anti-rabbit IgG secondary antibody (Proteintech, China; 1:5,000) for 1 h at room temperature. Signals were developed using ECL reagents (Beyotime, China) and captured on a chemiluminescence imaging system (Tanon, China). Band intensities were quantified by densitometry (ImageJ) and normalized to GAPDH. Unless stated otherwise, all blots were performed from ≥3 independent experiments. Statistical analysis of normalized densitometry used one-way ANOVA with Tukey’s post-hoc test; p < 0.05 was considered significant.

### 2.9 *In-vivo* antitumor studies

Male BALB/c nude mice (6–8 weeks; ∼18 g) were inoculated s. c. With 1 × 10^7^ PANC-1 cells. When tumors reached ∼60 mm^3^, mice were randomized to four groups: PBS, AV3, AV3+KH3, and AKNPs. Mice received intravenous injections of AV3 (2 mg/kg), AV3+KH3 (2 mg/kg + 2 mg/kg), or AKNPs at equivalent AV3/KH3 doses, administered every 2 days for a total of four treatments. After the last treatment, tumors were collected for H&E and Western blot analysis.

### 2.10 *In vivo* fluorescence imaging and biodistribution

To evaluate the *in vivo* biodistribution and tumor accumulation of AKNPs, Cy5-labeled AKNPs were intravenously injected into PANC-1 tumor–bearing BALB/c nude mice when tumor volumes reached approximately 100 mm^3^. Whole-body fluorescence imaging was performed at 2 h and 24 h post-injection using an *in vivo* imaging system (IVIS). During imaging, mice were anesthetized, and Cy5 fluorescence signals were acquired using appropriate excitation and emission filter settings (Ex ≈ 640 nm, Em ≈ 680 nm). All images were collected using identical exposure parameters to allow quantitative comparison.

At 24 h post-injection, mice were euthanized, and major organs (heart, liver, spleen, lung, and kidney) as well as tumors were harvested for *ex vivo* fluorescence imaging under the same acquisition conditions. Fluorescence intensity was quantified by region-of-interest (ROI) analysis and expressed as radiant efficiency (*p*/*s*/*cm* ^2^/*sr*)/(μW/cm^2^), enabling assessment of nanoparticle biodistribution and tumor accumulation.

### 2.11 TUNEL staining assay

Tumor tissues were harvested at the end of treatment, fixed in 4% paraformaldehyde, embedded in paraffin, and sectioned (4–5 μm). Apoptotic cells in tumor sections were detected using a TUNEL apoptosis detection kit according to the manufacturer’s instructions. Fluorescence images were acquired using a fluorescence microscope or confocal laser scanning microscope under identical imaging settings across groups. TUNEL-positive cells were visualized in the red channel, and nuclei were visualized in the blue channel.

### 2.12 Histological analysis of major organs

At the end of the *in vivo* treatment, major organs including the heart, liver, spleen, lung, and kidney were harvested from PANC-1 tumor-bearing mice and immediately fixed in 4% paraformaldehyde. After fixation, tissues were dehydrated through a graded ethanol series, cleared in xylene, and embedded in paraffin. Paraffin-embedded tissues were sectioned at a thickness of 4–5 μm and mounted on glass slides. Sections were then deparaffinized, rehydrated, and stained with hematoxylin and eosin (H&E) following standard histological procedures. Stained sections were imaged using a light microscope under identical settings across all groups to evaluate potential histopathological changes.

### 2.13 Serum biochemical analysis

At the end of the treatment period, blood samples were collected from mice via orbital sinus or cardiac puncture under anesthesia and allowed to clot at room temperature. Serum was obtained by centrifugation at 3,000 rpm for 10 min and stored at 4 °C until analysis. Serum biochemical parameters, including alanine aminotransferase (ALT), aspartate aminotransferase (AST), blood urea nitrogen (BUN), and creatinine (Cr), were measured using commercial assay kits according to the manufacturers’ protocols. All measurements were performed in triplicate using an automated biochemical analyzer. Data are presented as mean ± SD (n = 3), and statistical analysis was conducted using one-way analysis of variance (ANOVA), with p < 0.05 considered statistically significant.

### 2.14 Statistics

Data are mean ± SD. Two-group comparisons used two-tailed t-tests; multiple groups used one-way ANOVA. Significance was set at p < 0.05 (Graphpad).

## 3 Results

### 3.1 Clinical relevance of ITGA5 and PGAM1 in pancreatic cancer

**FIGURE 1:**
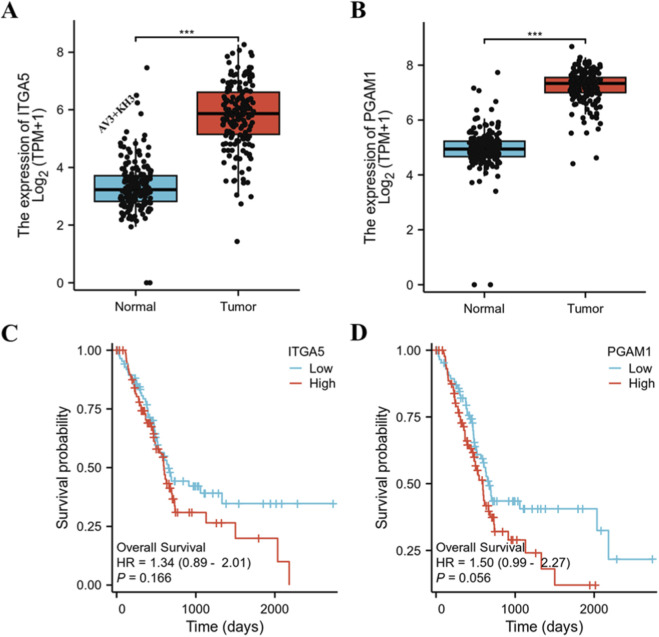
Expression and prognostic value of ITGA5 and PGAM1 in pancreatic cancer. **(A,B)** mRNA expression of ITGA5 (target of AV3) and PGAM1 (target of KH3) in normal pancreatic tissues (GTEx) and pancreatic cancer tissues (TCGA-PAAD) (Wilcoxon rank sum test, ***p ≤ 0.001). **(C,D)** Kaplan–Meier overall survival analysis based on ITGA5 and PGAM1 expression in TCGA-PAAD patients.

To evaluate the clinical significance of the molecular targets of AV3 and KH3, we analyzed the expression and prognostic value of ITGA5 and PGAM1 in pancreatic cancer patient datasets. Transcriptome analysis based on TCGA-PAAD and GTEx cohorts demonstrated that both ITGA5 and PGAM1 were significantly upregulated in pancreatic tumor tissues compared with normal pancreatic tissues (p ≤ 0.001) (Figures 1A,B). Kaplan–Meier survival analysis further revealed that patients with high ITGA5 or PGAM1 expression exhibited worse overall survival than those with low expression, consistent with their tumor-promoting roles (Figures 1C,D). Although the hazard ratios did not reach strict statistical significance (ITGA5: HR = 1.34, p = 0.166; PGAM1: HR = 1.50, p = 0.056), both markers showed a strong trend toward poor prognosis. These findings highlight the clinical relevance of targeting these pathways in pancreatic cancer therapy.

Based on these insights, we next sought to design a nanoparticle system that can simultaneously target ITGA5 and PGAM1 by co-delivering AV3 and KH3, thereby maximizing therapeutic efficacy.

### 3.2 Polymer and nanoparticle preparation

**FIGURE 2:**
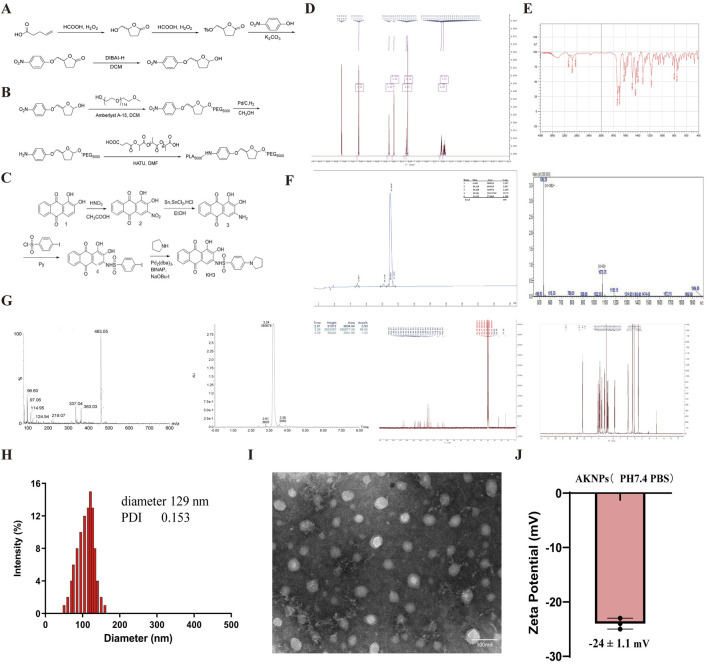
Synthesis and characterization of PLA–THF–PEG/AV3/KH3 nanoparticles (AKNPs) for simultaneous targeting of ITGA5 and PGAM1. **(A)** Synthetic route from 4-pentenoic acid to the THF derivative (p-nitrophenoxy-substituted lactol, brown solid). **(B)** Conversion of the THF derivative into the amphiphilic triblock copolymer PLA_5000_–THF–PEG_5000_. **(C)** Synthetic pathway of the KH3 small molecule from anthraquinone derivatives. **(D)** 1H NMR spectrum of the THF derivative. **(E)** FT-IR and GPC analyses confirming the structure and molecular weight distribution of PLA_5000_–THF–PEG_5000_. **(F)** HPLC and 1H NMR characterization of AV3 peptide. **(G)** MS and 1H NMR spectra confirming the structure of KH3. **(H)** Particle size distribution of PLA–THF–PEG/AV3/KH3 nanoparticles measured by DLS (average diameter ∼129 nm, PDI = 0.153). **(I)** TEM image showing spherical morphology and homogeneous dispersion of PLA–THF–PEG/AV3/KH3 nanoparticles. Scale bar = 100 nm **(J)** Zeta potential of PLA-THF-PEG/AV3/KH3 nanoparticles measured in aqueous solution.

To construct a nanoparticle system capable of simultaneously targeting ITGA5 and PGAM1, we employed the amphiphilic copolymer PLA–THF–PEG as the delivery scaffold, and co-encapsulated AV3 (ITGA5 inhibitor peptide) and KH3 (PGAM1 small-molecule inhibitor) to form PLA-THF-PEG/AV3/KH3 nanoparticles (AKNPs). The amphiphilic copolymer PLA-THF-PEG was synthesized through a multi-step strategy starting from 4-pentenoic acid (Figure 2A). The terminal double bond of 4-pentenoic acid was oxidized with performic acid to generate the corresponding epoxide, which was subsequently tosylated and substituted with p-nitrophenol. Selective reduction with DIBAL-H yielded a THF derivative (brown solid, p-nitrophenoxy-substituted lactol), serving as the key intermediate. This THF derivative was conjugated with PEG_5000_ under Amberlyst A-15 catalysis and further coupled with succinic anhydride–modified PLA_5000_ using HATU-mediated amidation, resulting in the final amphiphilic copolymer PLA_5000_–THF–PEG_5000_ (Figure 2B).

In parallel, the small molecule KH3 was synthesized from anthraquinone derivatives through nitration, reduction, sulfonylation, and Pd-catalyzed coupling (Figure 2C), while the AV3 peptide (Ac-RYYRITY) was obtained by Fmoc-based solid-phase synthesis.

The structures of all intermediates and final products were confirmed by spectroscopic analysis. ^1H^ NMR confirmed the THF derivative (Figure 2D), while FT-IR and GPC analysis verified the successful preparation and uniform molecular weight distribution of PLA_5000_–THF–PEG_5000_ (Figure 2E; Supplementary Figure S7). Polystyrene-calibrated GPC revealed a number-average molecular weight (Mn) of 10,860 g mol^-1^ and a narrow dispersity (Mw/Mn) of 1.11, consistent with the formation of a well-defined block copolymer. AV3 was validated by HPLC and ^1H^ NMR (Figure 2F), and KH3 was confirmed by MS and ^1H^ NMR (Figure 2G).

Using the synthesized PLA-THF-PEG copolymer as a carrier, PLA-THF-PEG/AV3/KH3 nanoparticles were prepared by nanoprecipitation. The copolymer, AV3, and KH3 were co-dissolved in organic solvent and slowly introduced into aqueous solution, followed by dialysis to remove residual solvent. Dynamic light scattering (DLS) analysis revealed an average particle size of 129 nm with a narrow distribution (PDI = 0.153) (Figure 2H). Transmission electron microscopy (TEM) further confirmed that the nanoparticles exhibited a spherical morphology with homogeneous dispersion in aqueous solution (Figure 2I). In addition, zeta-potential measurements showed that AKNPs exhibited a surface charge of −24.1 ± 1.1 mV in aqueous media, indicating favorable colloidal stability (Figure 2J). Quantitative analysis further demonstrated efficient drug encapsulation within AKNPs, with encapsulation efficiencies (EE%) of 58.11% ± 1.52% for KH3 and 85.87% ± 2.55% for AV3, and corresponding drug loading contents (DL%) of 12.6% ± 0.4% and 21.8% ± 0.7%, respectively (Table 1).

### 3.3 pH-responsive release and colloidal stability of nanoparticles

**FIGURE 3:**
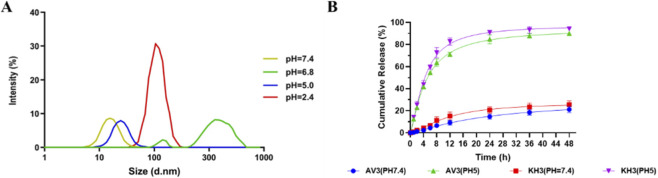
pH-responsive colloidal behavior and drug release profiles of PLA–THF–PEG/AV3/KH3 nanoparticles. **(A)** Dynamic light scattering (DLS) size distribution profiles of nanoparticles incubated at different pH values (pH 7.4, 6.8, 5.0, and 2.4). **(B)** *In vitro* cumulative release profiles of AV3 and KH3 from PLA–THF–PEG nanoparticles under different pH conditions at various time points.

To evaluate the acid responsiveness of the nanocarrier, drug-loaded PLA–THF–PEG/AV3/KH3 nanoparticles were incubated in media of different pH values (7.4, 6.8, 5.0, and 2.4) and their hydrodynamic sizes were monitored by DLS (Figure 3A). At physiological pH (7.4), nanoparticles displayed a narrow peak around ∼20 nm, indicating excellent colloidal stability under neutral conditions. At pH 6.8, however, a broad peak centered at ∼300 nm appeared, suggesting partial aggregation due to incomplete cleavage of the acid-labile THF linkages and disruption of hydrophilic/hydrophobic balance. Interestingly, at pH 5.0 the size distribution reverted to a smaller peak (∼20–30 nm), indicating that stronger acidity promoted more complete cleavage of acid-sensitive bonds, leading to nanoparticle disassembly into smaller and more stable fragments. At pH 2.4, a sharp peak appeared around ∼120 nm, consistent with significant structural rearrangement and destabilization of the carrier.

Together, these results demonstrate that PLA–THF–PEG/AV3/KH3 nanoparticles (AKNPs) are stable under neutral conditions, undergo partial aggregation under mildly acidic environments (pH ∼6.8, typical of tumor extracellular milieu), and disassemble into smaller fragments under strongly acidic conditions (pH ≤ 5.0, representative of endosomal/lysosomal compartments). This pH-responsive colloidal transformation highlights their potential for site-specific drug release in the tumor microenvironment and intracellular acidic organelles.

Consistent with the observed pH-dependent colloidal behavior, the *in vitro* release profiles of AV3 and KH3 from AKNPs further confirmed their acid-responsive characteristics (Figure 3B). Under physiological conditions (pH 7.4), both AV3 and KH3 exhibited slow and limited release, with cumulative release remaining below ∼25% within 48 h, indicating good stability and minimal premature drug leakage during systemic circulation. In contrast, under acidic conditions (pH 5.0), mimicking the endo/lysosomal environment, AKNPs showed rapid and sustained drug release, with cumulative release reaching approximately ∼85% for AV3 and ∼95% for KH3 within 48 h. This accelerated release is attributed to the acid-triggered cleavage of the THF linkages, leading to nanocarrier disassembly and efficient drug liberation.

In addition to pH responsiveness, the colloidal stability of AKNPs in biologically relevant media was further evaluated by incubating the nanoparticles in phosphate-buffered saline (PBS, pH 7.4) and serum-containing medium for up to 72 h (Supplementary Figure S5). In PBS, AKNPs predominantly maintained small hydrodynamic sizes (∼20–40 nm) with only a slight size increase over time, indicating good stability under buffer conditions. In contrast, incubation in serum-containing medium resulted in an initial moderate increase in particle size, followed by the appearance of larger populations or bimodal size distributions (∼40–200 nm) at later time points, consistent with serum protein adsorption and protein-mediated bridging or aggregation effects.

### 3.4 Cellular uptake and tumor cell–targeting ability of AKNPs

**FIGURE 4:**
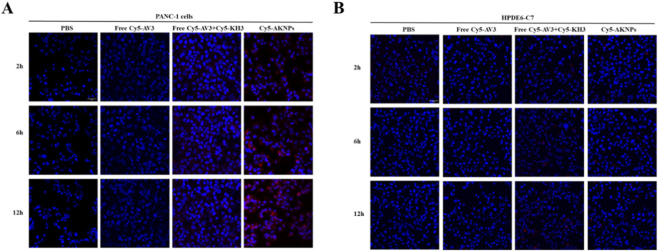
*In vitro* tumor cell–targeting and cellular uptake of AKNPs. **(A)** Representative CLSM images of tumor cells treated with PBS, free AV3, free AV3+KH3,AKNPs **(B)** Representative CLSM images of normal cells treated under identical conditions.

The cellular uptake and tumor cell–targeting capability of AKNPs were investigated by confocal laser scanning microscopy (CLSM) using Cy5-labeled nanoparticles. Tumor cells (PANC-1) and normal pancreatic duct epithelial cells (HPDEC) were incubated with PBS, free AV3, free AV3+KH3, or Cy5-labeled AKNPs under identical conditions. As shown in Figure 4A, PANC-1 cells treated with PBS or free drugs exhibited only blue nuclear staining (DAPI) and negligible red fluorescence, indicating minimal nonspecific uptake. In contrast, cells treated with Cy5-AKNPs showed strong and time-dependent intracellular red fluorescence, which increased markedly from early to later incubation time points. The Cy5 signals were predominantly localized in the cytoplasmic region and showed clear co-localization with cellular structures, confirming efficient internalization of AKNPs by tumor cells. In comparison, uptake studies performed in HPDEC normal cells revealed a markedly different pattern (Figure 4B). Under the same treatment conditions, Cy5-AKNPs produced minimal intracellular fluorescence in HPDEC cells, even at extended incubation times, indicating substantially lower nanoparticle internalization in non-malignant cells. Together, these results demonstrate that AKNPs exhibit preferential uptake by pancreatic cancer cells over normal pancreatic epithelial cells, supporting their tumor cell–targeting capability *in vitro*.

### 3.5 *In vitro* efficacy and mechanistic markers

The cytotoxicity of AV3, AV3+KH3, or AKNPs, was evaluated in PANC-1 cells using CCK-8 assays. AV3 showed moderate inhibitory effects, with significant reduction of cell viability at concentrations above 40 μM (Supplementary Figure S1A), whereas KH3 exhibited potent inhibition near 2 μM (Supplementary Figure S1B). Notably, the pH-responsive AKNPs achieved substantially stronger cytotoxicity than either free drug alone ((Supplementary Figure S2A), underscoring the synergistic effect of dual delivery and the advantage of tumor-selective release. Consistent with these findings, Annexin V-FITC/PI flow cytometry analysis further demonstrated that AKNPs treatment induced higher proportion of apoptotic cells compared with free AV3 or AV3 + KH3 (Supplementary Figures S2B,S2C), providing additional qualitative and quantitative evidence of the enhanced *in vitro* therapeutic efficacy of the combined nanoparticle formulation.

**FIGURE 5:**
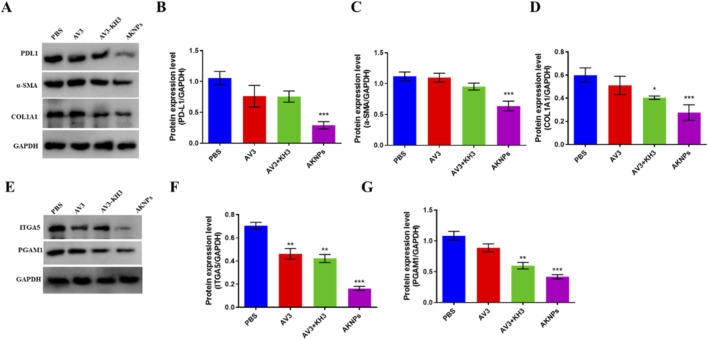
Western blot analysis of PANC-1 cells treated with PBS, AV3, AV3+KH3, or AKNPs. **(A)** Western blot analysis of fibrosis-related proteins (α-SMA, COL1A1), immune checkpoint protein (PD-L1), in PANC-1 cells after different treatments (PBS, AV3, AV3+KH3, AKNPs). GAPDH served as the loading control. **(B–D)** Quantification of PD-L1, α-SMA, and COL1A1 relative to GAPDH. **(E)** Western blot analysis of functional targets of AV3 (ITGA5) and KH3 (PGAM1) in tumor tissues after different treatments (PBS, AV3, AV3+KH3, AKNPs). GAPDH served as the loading control. **(F,G)** Quantification of ITG5, and PGAM1 relative to GAPDH. Data are presented as mean ± SD (n = 3). Statistical analysis was performed using Student’s t-test; *p ≤ 0.05, **p ≤ 0.01, ***p ≤ 0.001.

Mechanistic investigations were performed via Western blot analysis (Figure 5). Treatment with AKNPs resulted in greatly downregulation of fibrosis-related proteins α-SMA and COL1A1, indicating suppression of tumor-associated fibroblast activation and desmoplastic reaction. In addition, ITGA5 expression was specifically reduced by AV3-containing groups, while PGAM1 levels were significantly suppressed by KH3 treatment. Importantly, PD-L1 expression, an immune checkpoint marker, was also decreased in the nanoparticle group, suggesting enhanced tumor immunogenicity.

### 3.6 *In vivo* antitumor activity

**FIGURE 6:**
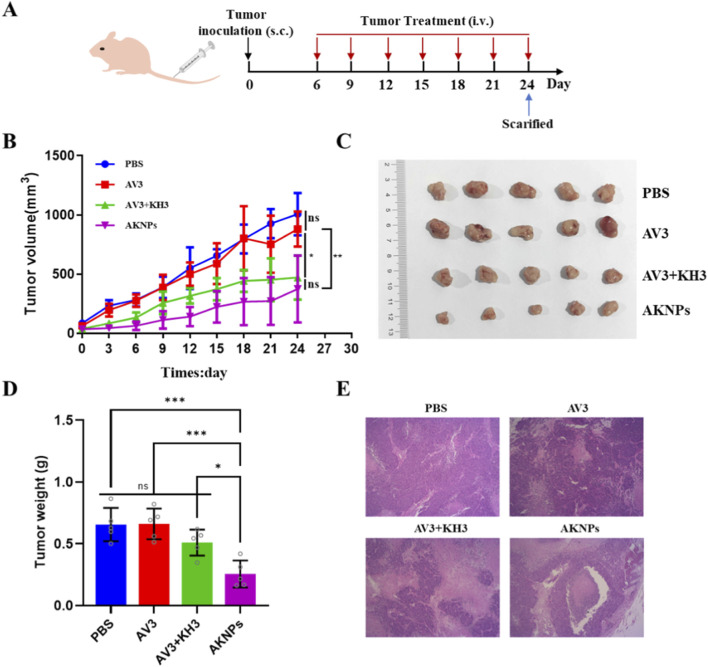
*In vivo* antitumor efficacy of AKNPs in PANC-1 xenograft model. **(A)** Schematic illustration of the treatment schedule. **(B)** Tumor growth curves of BALB/c nude mice treated with PBS, free AV3, free AV3+KH3, or AKNPs. **(C)** Representative images of tumors harvested at the end of treatment. **(D)** Tumor weights of each group. **(E)** Representative H&E staining of tumor tissues from different treatment groups. Data are presented as mean ± SD (n = 5). Statistical analysis was performed using one-way ANOVA; **p ≤ 0.01, ***p ≤ 0.001.

The therapeutic efficacy of AKNPs was further validated in a PANC-1 xenograft mouse model. Tumor-bearing BABL/c nude mice were treated via intravenous injection with PBS, free AV3, free AV3+KH3, or AKNPs. Tumor volume measurements over the treatment period (Figure 6A). Tumor volumes in the PBS group increased rapidly throughout the study, reaching over 1,000 mm^3^ by day 24. Treatment with free AV3 slightly delayed tumor growth compared with PBS, but the difference was not statistically significant (ns). The AV3+KH3 combination group exhibited a moderate reduction in tumor growth, with tumor volumes lower than PBS or AV3 groups. In contrast, mice treated with AKNPs displayed the most significant tumor growth inhibition, with final tumor volumes significantly smaller than those of all other groups (Figure 6B).

Individual tumor growth curves for each mouse are presented in Supplementary Figure S3, further illustrating the consistent tumor growth suppression observed in the AKNP-treated group compared with the control and free drug groups. These results indicate that encapsulation of AV3 and KH3 into the pH-responsive nanoparticle formulation markedly enhances their antitumor efficacy compared with free drugs. In addition to quantitative tumor growth measurements, representative images of tumor-bearing mice at the end of treatment are shown in Supplementary Figure S4.

At the endpoint of treatment, tumors excised from the AKNP group were visibly smaller in size (Figure 6C) and significantly lighter in weight (Figure 6D) compared with PBS, AV3, or AV3+KH3 groups, further confirming enhanced therapeutic efficacy. Histopathological analysis of tumor tissues by H&E staining (Figure 6E) revealed reduced tumor cell density, stromal content, and increased necrotic areas in the AKNP group, in contrast to the denser tumor architecture observed in the PBS and free drug groups.

TUNEL staining was performed to evaluate tumor cell apoptosis *in vivo*. As shown in Supplementary Figure S6, minimal apoptotic signals in tumors from the PBS group, whereas increased TUNEL-positive staining was observed after AV3 or AV3+KH3 treatment. In contrast, tumors treated with AKNPs exhibited markedly enhanced TUNEL-positive signals throughout the tumor tissue, indicating stronger induction of tumor cell apoptosis *in vivo*.

**FIGURE 7:**
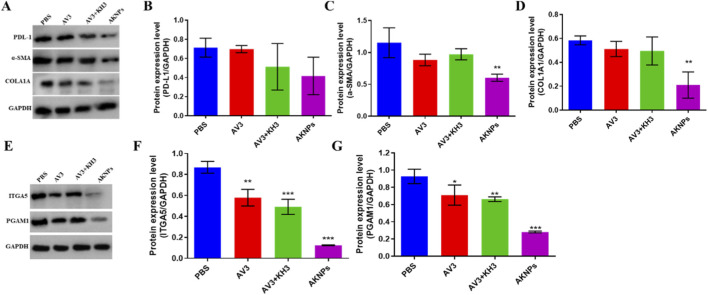
Western blot analysis of tumor tissues from PANC-1 xenograft mice. **(A)** Western blot bands of fibrosis-related proteins (α-SMA, COL1A1), immune checkpoint protein (PD-L1), in tumor tissues after different treatments (PBS, AV3, AV3+KH3, AKNPs). GAPDH served as the loading control. **(B–D)** Quantitative analysis of protein expression levels of PD-L1, α-SMA, and COL1A1 normalized to GAPDH. **(E)** Western blot bands of functional targets of AV3 (ITGA5) and KH3 (PGAM1) in tumor tissues after different treatments (PBS, AV3, AV3+KH3, AKNPs). GAPDH served as the loading control. **(F,G)** Quantitative analysis of protein expression levels of ITGA5 and PGAM1 normalized to GAPDH. Data are presented as mean ± SD (n = 3). Statistical analysis was performed using Student’s t-test; *p ≤ 0.05, **p ≤ 0.01, ***p ≤ 0.001.

To elucidate the molecular mechanisms underlying the superior antitumor efficacy of AKNPs observed *in vivo*, we further analyzed protein expression in tumor tissues by Western blotting (Figure 7). Compared with the PBS group, AKNPs markedly reduced the expression of fibrosis-associated proteins α-SMA and COL1A1 as well as the immune checkpoint protein PD-L1, indicating strong inhibition of stromal remodeling and improvement of immune sensitivity in the tumor microenvironment. Moreover, AV3-containing groups significantly downregulated ITGA5, consistent with its role as an integrin α5 antagonist, while KH3-containing groups suppressed PGAM1 expression, validating the glycolytic regulation activity of KH3. Importantly, the AKNPs group exhibited the most significant downregulation across all targets, reflecting the synergistic effects of dual-drug loading and pH-responsive release. These results highlight that AKNPs not only suppress tumor growth but also remodel the desmoplastic stroma and sensitize tumors to immune modulation.

### 3.7 *In vivo* biodistribution and tumor-targeting ability of AKNPs

**FIGURE 8:**
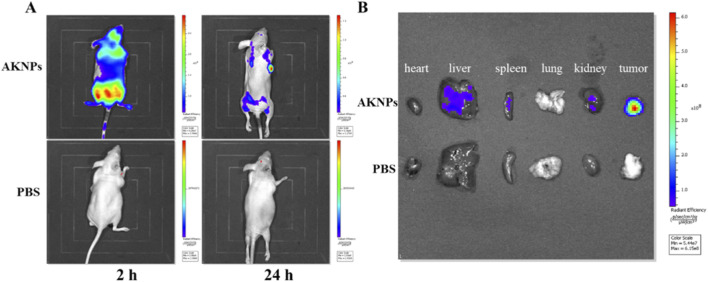
*In vivo* biodistribution and tumor-targeting ability of AKNPs. **(A)** Representative *in vivo* fluorescence images of PANC-1 tumor–bearing nude mice after intravenous administration of Cy5-labeled AKNPs. **(B)** *Ex vivo* fluorescence imaging of major organs (heart, liver, spleen, lung, kidney) and tumor harvested after imaging. Fluorescence intensity is presented as radiant efficiency (p/s/cm^2^/sr)/(μW/cm^2^).

The *in vivo* biodistribution and tumor-targeting ability of AKNPs were evaluated using near-infrared fluorescence imaging in PANC-1 tumor-bearing nude mice after intravenous administration of Cy5-labeled AKNPs. As shown in Figure 8A, whole-body fluorescence imaging revealed a time-dependent distribution pattern of AKNPs. Following systemic injection, fluorescence signals were initially observed throughout the body, followed by progressive accumulation at the tumor site over time. The tumor-associated fluorescence became increasingly prominent at later time points, indicating effective tumor retention of AKNPs.

*Ex vivo* fluorescence imaging of excised organs further confirmed the *in vivo* observations (Figure 8B). Compared with major organs including the heart, spleen, lung, and kidney, the tumor tissue exhibited markedly stronger fluorescence intensity.

### 3.8 Safety evaluation of AKNPs *in vivo*

**FIGURE 9:**
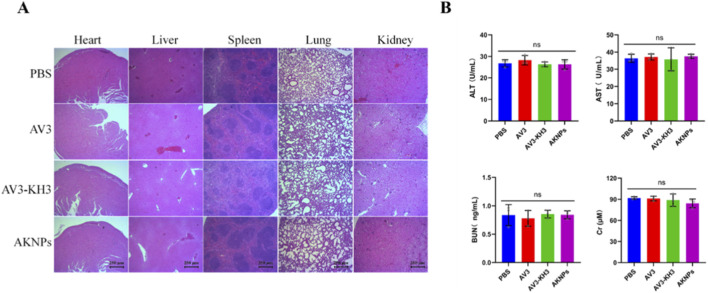
*In vivo* safety evaluation of AKNPs. **(A)** Representative hematoxylin and eosin (H&E)–stained sections of major organs (heart, liver, spleen, lung, and kidney) collected from PANC-1 tumor–bearing mice after treatment with PBS, AV3, AV3+KH3, or AKNPs. Scale bar = 250 μm. **(B)** Serum biochemical analysis of hepatic and renal function, including alanine aminotransferase (ALT), aspartate aminotransferase (AST), blood urea nitrogen (BUN), and creatinine (Cr), after different treatments. Data are presented as mean ± SD (n = 3). Statistical analysis was performed using one-way ANOVA; ns, not significant.

The *in vivo* biosafety of AKNPs was evaluated by histological examination of major organs and serum biochemical analysis in PANC-1 tumor–bearing mice. After the completion of treatment, major organs including the heart, liver, spleen, lung, and kidney were harvested and subjected to hematoxylin and eosin (H&E) staining. As shown in Figure 9A, no obvious pathological abnormalities, inflammatory infiltration, tissue necrosis, or structural damage were observed in any of the examined organs from mice treated with AKNPs, AV3, or AV3+KH3, compared with the PBS control group. The overall tissue architecture of all major organs remained intact, indicating the absence of apparent treatment-related toxicity. In addition, serum biochemical parameters associated with hepatic and renal function were analyzed, including alanine aminotransferase (ALT), aspartate aminotransferase (AST), blood urea nitrogen (BUN), and creatinine (Cr). As shown in Figure 9B, no significant differences were detected among the PBS, AV3, AV3+KH3, and AKNP-treated groups (ns), suggesting that AKNP administration did not induce detectable liver or kidney dysfunction.

## 4 Discussion

Recent advances in nanomedicine have highlighted multifunctional delivery systems as promising tools to overcome the therapeutic barriers of PDAC. Traditional single-target therapies often fail due to tumor heterogeneity and adaptive signaling, whereas dual- or multi-target nanoplatforms provide opportunities to remodel the stroma, interfere with metabolism, and enhance immune responses (Peng et al., 2025; Pontón and Sánchez-García, 2025). In particular, pH-responsive carriers have attracted considerable attention, as they exploit the acidic tumor microenvironment to enable site-specific drug release and reduce systemic toxicity (Sabit et al., 2025; Liu et al., 2025; Shabnum et al., 2024). These developments provide the rationale for integrating stromal, metabolic, and immune modulation into a unified therapeutic system.

In this study, we developed a pH-responsive nanoplatform (PLA-THF-PEG/AV3/KH3, AKNPs) that co-delivers AV3 and KH3 to simultaneously target ITGA5-mediated stromal activation and PGAM1-driven metabolic reprogramming in pancreatic cancer. AKNPs exhibited excellent colloidal stability under physiological conditions and triggered site-specific drug release in acidic tumor-associated environments. Both *in vitro* and *in vivo* experiments demonstrated that AKNPs not only enhanced cytotoxic efficacy but also remodeled the desmoplastic stroma, suppressed fibrosis-related and immune checkpoint proteins, and improved tumor immunogenicity.

Although this study demonstrates that AKNPs exert synergistic antitumor effects by modulating stroma, metabolism, and immunity, several limitations remain. The *in vivo* validation was restricted to a subcutaneous PANC-1 xenograft, which lacks the complex stromal and immune features of PDAC; orthotopic mouse models would provide greater relevance. PDAC is also highly heterogeneous, and while ITGA5 and PGAM1 were used as representative targets, other stromal and metabolic mediators may contribute to progression and therapy resistance, highlighting the need for broader pathway exploration or combination with immunotherapies such as PD-1/PD-L1 blockade (Thakur et al., 2025; Wu et al., 2024; Farhangnia et al., 2024). Future studies employing humanized mouse models will be required to fully elucidate the immune-evasion properties, tumor immune microenvironment remodeling, and potential synergy with immunotherapy. The pharmacokinetics, biodistribution, and long-term biosafety of AKNPs require further study despite the absence of overt toxicity in our experiments (Tempero et al., 2021). In addition, the clinical significance of ITGA5 and PGAM1 was inferred from retrospective datasets, and validation in clinical samples is necessary.

Together, these findings highlight a synergistic strategy that integrates stromal remodeling, metabolic interference, and immune modulation into a single therapeutic system. AKNPs represent a promising nanomedicine platform for overcoming the therapeutic barriers of PDAC and may offer translational potential for future combination with immunotherapies.

## 5 Conclusion

We developed pH-sensitive PLA–THF–PEG nanoparticles (AKNPs) that co-deliver AV3 (ITGA5 antagonist) and KH3 (PGAM1 inhibitor) with acid-triggered release, achieving enhanced cellular uptake, potent cytotoxicity, and concurrent suppression of α-SMA, COL1A1, ITGA5, and PGAM1. In PANC-1 xenografts, AKNPs significantly reduced tumor growth and stromal fibrosis, outperforming single agents and their free combination. These findings demonstrate that dual-targeted, pH-responsive co-delivery can overcome key stromal and metabolic barriers in PDAC and improve therapeutic efficacy. Future work will optimize dosing and release kinetics, define pharmacokinetics/pharmacodynamics and long-term safety, and evaluate efficacy in orthotopic and immunocompetent models, including combinations with immune checkpoint blockade.

## Data Availability

The original contributions presented in the study are included in the article/Supplementary Material, further inquiries can be directed to the corresponding authors.
