# Supplementary material for: PLA-THF-PEG nanoparticles Co-encapsulating AV3 and KH3 for synergistic pancreatic cancer therapy via stromal remodeling and metabolic inhibition
Source: Front Pharmacol. 2026 Feb 4;17:1723694. doi: 10.3389/fphar.2026.1723694 (PMC12913430; doi:10.3389/fphar.2026.1723694)
Supplement: Supplementary file 1 [file Supplementaryfile1.docx]

Supplement Materials


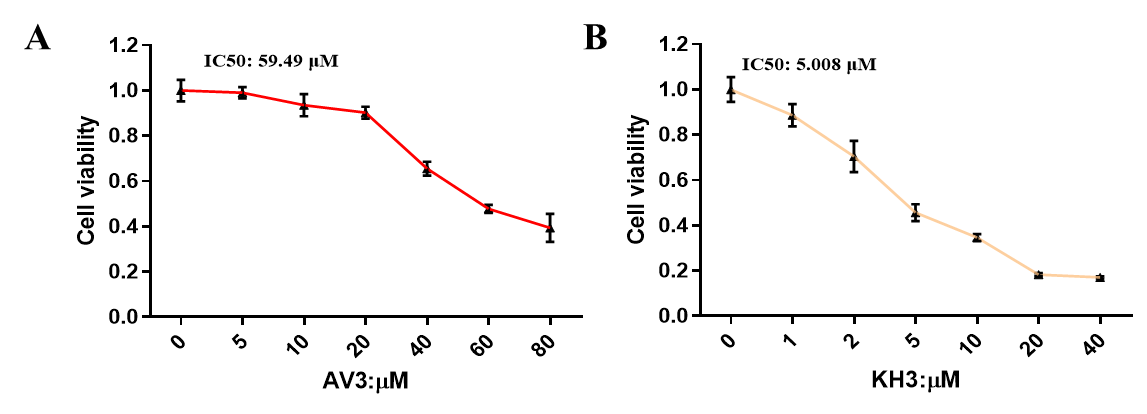


Figure S1. CCK-8 assay evaluating cytotoxicity of AV3 and KH3 in PANC-1 cells.

PANC-1 cells were treated with increasing concentrations of AV3 (A) or KH3 (B) for 48 h, and cell viability was measured by CCK-8 assay. Data are presented as mean ± SD (n = 3). Statistical analysis was performed using Student’s t-test, *p ≤ 0.05, **p ≤ 0.01, ***p ≤ 0.001.


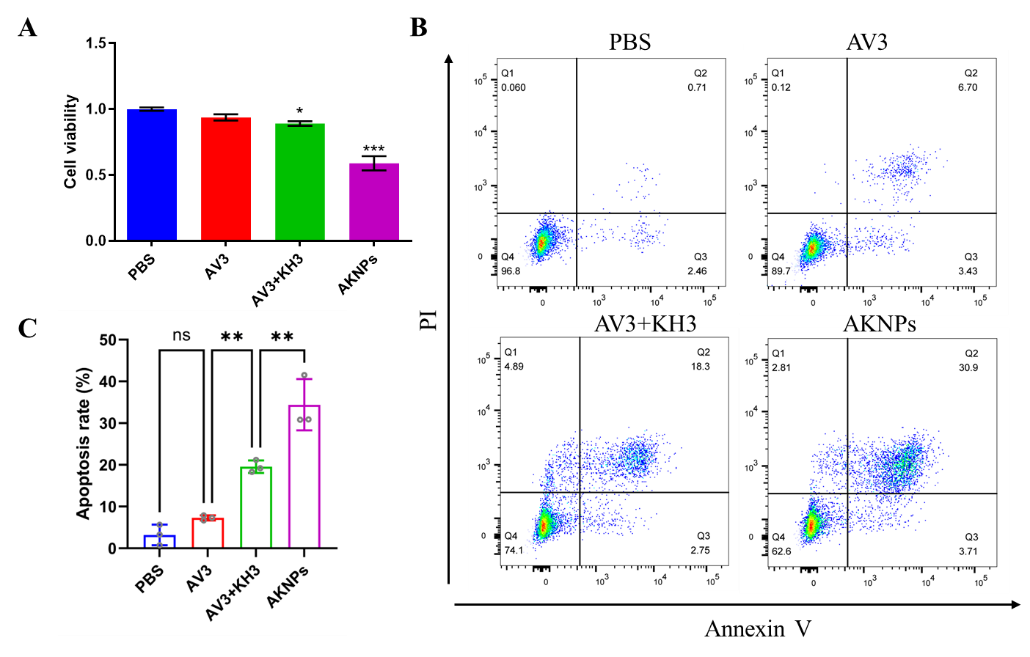


Figure S2. Effects of AV3, AV3 + KH3, and AKNPs on cell viability and apoptosis in PANC-1 cells. (A) CCK-8 assay of PANC-1 cells after 48 h treatment with PBS, free AV3, free AV3+KH3, or pH-responsive nanoparticles (AKNPs). (B) Representative Annexin V-FITC/PI flow cytometry dot plots of PANC-1 cells following the indicated treatments. (C) Quantitative analysis of apoptosis rates derived from the flow cytometry data shown in (B). Data are presented as mean ± SD. ns, not significant; **P < 0.01.


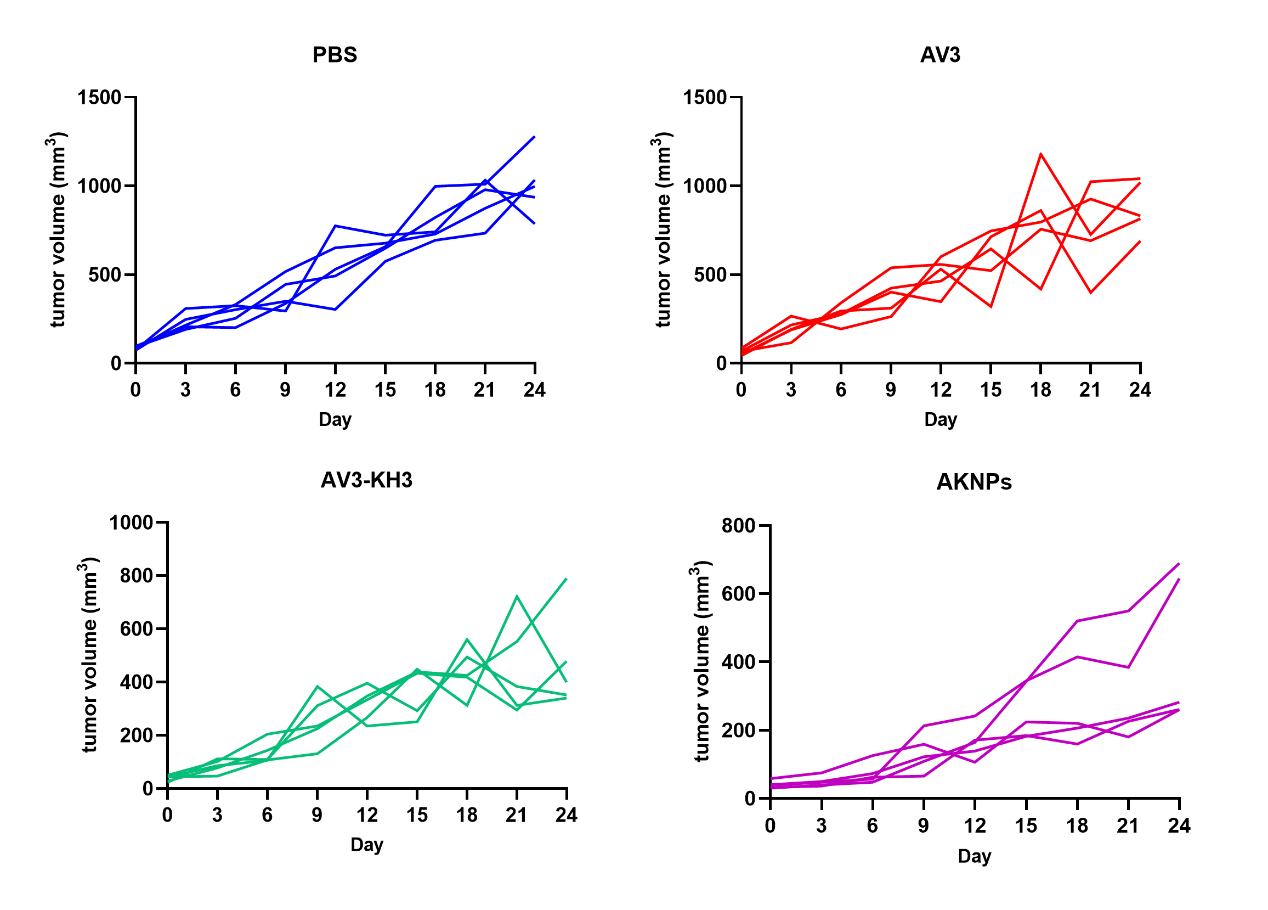


Figure S3. Individual tumor growth curves in PANC-1 xenograft-bearing mice.

Tumor volumes of individual mice (n = 5 per group) treated with PBS, free AV3, free AV3+KH3, or AKNPs were monitored over 24 days. Each line represents the tumor growth of one mouse.


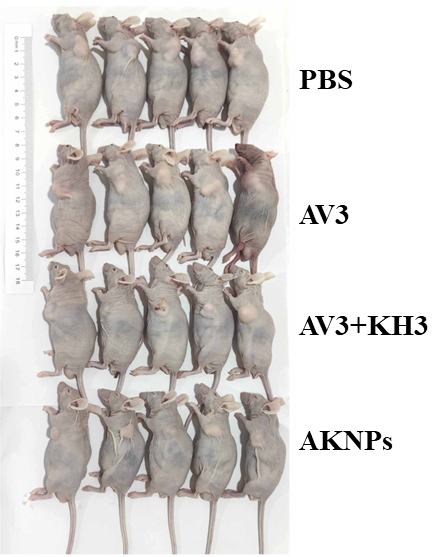


Figure S4. Representative images of tumor-bearing mice after treatment.

Photographs of PANC-1 xenograft-bearing BALB/c nude mice (n = 5 per group) at the end of treatment with PBS, free AV3, free AV3+KH3, or AKNPs.


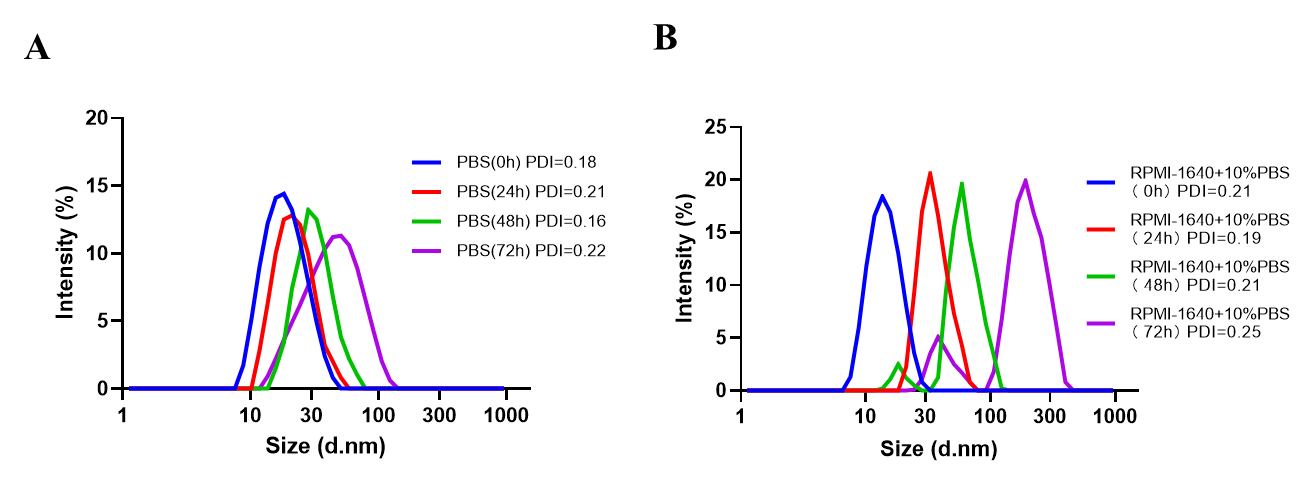


Figure S5. Colloidal stability of AKNPs in different media.

(A) Dynamic light scattering (DLS) size distribution profiles of AKNPs incubated in phosphate-buffered saline (PBS, pH 7.4) for up to 72 h. (B) DLS size distribution profiles of AKNPs incubated in serum-containing medium.


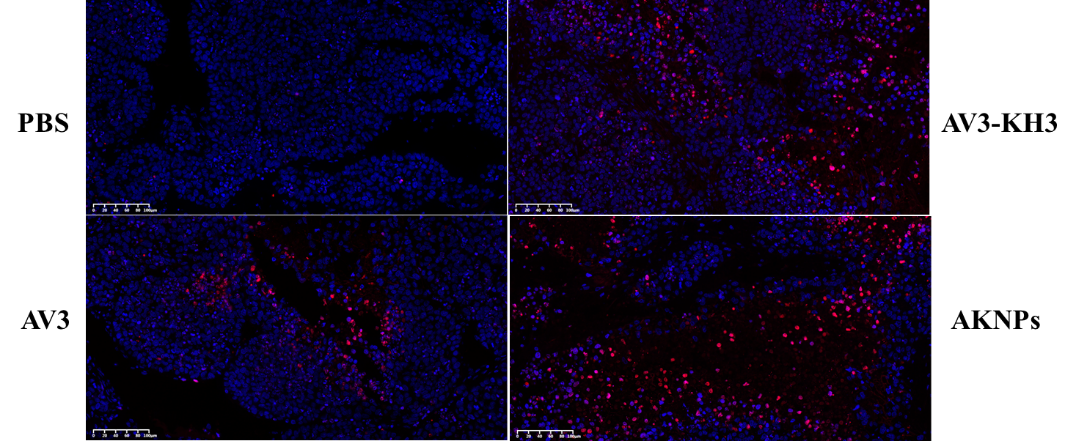


Figure S6. TUNEL staining analysis of tumor apoptosis in vivo.

Representative TUNEL staining images of tumor sections collected from PANC-1 tumor–bearing nude mice after different treatments. Apoptotic cells were detected by TUNEL staining (red), and cell nuclei were counterstained with DAPI (blue). Scale bar = 100 μm.


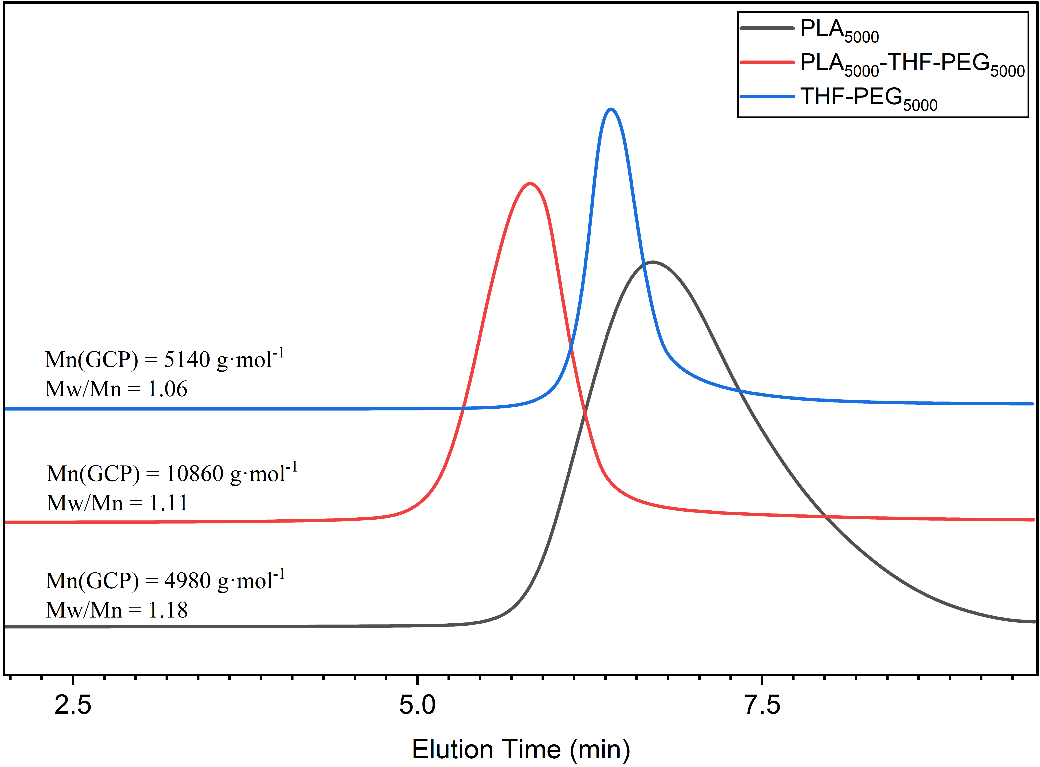


Figure S7. GPC elution curves of PLA_5000_, THF-PEG_5000_, and PLA_5000_-THF-PEG_5000_
